# Comprehensive analysis of the role of SFXN family in breast cancer

**DOI:** 10.1515/med-2023-0685

**Published:** 2023-04-01

**Authors:** Ding Yuan, Jialiang Liu, Wenbo Sang, Qing Li

**Affiliations:** Department of General Surgery, Shouguang City People’s Hospital, Shouguang, 262700, China

**Keywords:** sideroflexin, breast cancer, biomarkers, prognosis

## Abstract

The sideroflexin (SFXN) family is a group of mitochondrial membrane proteins. Although the function of the SFXN family in mitochondria has been widely recognized, the expression levels, role, and prognostic value of this family in breast cancer (BC) have not been clearly articulated and systematically analysed. In our research, SFXN1 and SFXN2 were significantly upregulated in BC versus normal samples based on Gene Expression Profiling Interactive Analysis 2 and the Human Protein Atlas databases. We found that high SFXN1 expression was significantly related to poor prognosis in BC patients and that high SFXN2 expression was significantly associated with good prognosis in BC patients. Gene Ontology analysis of the SFXN family was performed based on the STRING database to explore the potential functions of this family, including biological processes, cellular components, and molecular functions. Based on the MethSurv database, we found that two SFXN1 CpG sites (5′-UTR-S_Shelf-cg06573254 and TSS200-Island-cg17647431), two SFXN2 CpG sites (3′-UTR-Open_Sea-cg04774043 and Body-Open_Sea-cg18994254), one SFXN3 CpG site (Body-S_Shelf-cg17858697), and nine SFXN5 CpG sites (1stExon;5′-UTR-Island-cg03856450, Body-Open_Sea-cg04016113, Body-Open_Sea-cg04197631, Body-Open_Sea-cg07558704, Body-Open_Sea-cg08383863, Body-Open_Sea-cg10040131, Body-Open_Sea-cg10588340, Body-Open_Sea-cg17046766, and Body-Open_Sea-cg22830638) were significantly related to the prognosis of BC patients. According to the ENCORI database, four negative regulatory miRNAs for SFXN1 (hsa-miR-22-3p, hsa-miR-140-5p, hsa-miR-532-5p, and hsa-miR-582-3p) and four negative regulatory miRNAs for SFXN2 (hsa-miR-9-5p, hsa-miR-34a-5p, hsa-miR-532-5p, and hsa-miR-885-5p) were related to poor prognosis for BC patients. This study suggests that SFXN1 and SFXN2 are valuable biomarkers and treatment targets for patients with BC.

## 1 Introduction

Breast cancer (BC) is a common type of cancer in women and a major cause of cancer death, and the incidence of BC is increasing worldwide [1,2]. BC is caused by multiple factors (genetic, hormonal, and environmental); in particular, gene mutation plays an important role in the occurrence and development of BC [3,4]. At present, with the rise of personalized treatment for BC, both the oestrogen receptor and human epidermal growth factor receptor 2 have been confirmed as therapeutic targets for BC, and many BC patients benefit from them [5,6,7,8]. However, more markers need to be discovered for clinical application in the precise therapy of BC patients.

The members of the sideroflexin (SFXN) family are mitochondrial membrane proteins. The SFXN family has five members, namely, SFXN1, SFXN2, SFXN3, SFXN4, and SFXN5, which are classified in SLC56 [9]. SFXN1 acts as a mitochondrial serine transporter in single-carbon metabolism [10]. SFXN1 deficiency causes damage to the mitochondrial respiratory chain, compromising the biosynthesis, activity, and assembly of complex III (CIII) and reducing the level of coenzyme Q [11]. Mon et al. reported that SFXN2 is a mitochondrial outer membrane protein that plays a role in mitochondrial iron homeostasis by regulating haem biosynthesis [12]. Chen et al. reported that upregulated SFXN2 expression limits mitochondrial autophagy and increases iron-mediated energy production to promote the proliferation of multiple myeloma cells [13]. SFXN3 is an α-synuclein-dependent mitochondrial protein that regulates synaptic morphology and affects neurodegenerative pathways in humans [14,15]. SFXN4 is a complex I assembly factor, and SFXN4 deficiency causes macrocytic anaemia and mitochondrial disease [16,17]. Yoshikumi et al. reported that the expression level of SFXN5 in the pancreatic islets of rats with streptozotocin-induced diabetes was upregulated compared with that in normal rats [18]. However, although the role of the SFXN family in some cancers, such as lung adenocarcinoma and myeloma, has been studied in recent years [13,19], the expression and potential carcinogenic effects of the SFXN family in BC have not been clearly reported.

To elucidate the potential role of the SFXN family in BC, we performed systematic bioinformatics analysis of the SFXN family in BC, including expression patterns, functions, and prognostic value for BC patients. In addition, miRNAs that might regulate the expression of SFXN1 and SFXN2 in BC were found, and miRNA‒mRNA interaction had an impact on the prognosis of BC patients. This study advances the state of knowledge regarding the SFXN family in BC.

## 2 Materials and methods

### 2.1 Gene expression profiling interactive analysis 2 (GEPIA) analysis

GEPIA2 [20] (http://gepia2.cancer-pku.cn/) is a Web server that analyses RNA sequencing expression data from 9,736 tumours and 8,587 normal samples of TCGA and GTEx datasets using standard processing pipelines. We used GEPIA2 database to analyse the RNA expression level of SFXN family and ten nearest neighbour genes significantly related to SFXN1 and SFXN2 in BC samples. In addition, we explored the correlation among SFXN1–SFXN5 based on GEPIA2 database.

### 2.2 Human protein atlas (HPA) analysis

All data in the HPA [21] (https://www.proteinatlas.org/about) database is open access, allowing scientists in academia and industry to freely access the data to explore the human proteome. We detected the expression levels of SFXN1–SFXN5 in BC tissues compared with normal tissues. The antibodies used in the database were HPA063745, HPA018150, HPA008028, HPA020872, and HPA015473.

### 2.3 Survival analysis

Using Kaplan–Meier plotter [22] (http://www.kmplot.com/), we analysed the impact of SFXN family on the survival rate of patients with BC. Moreover, 1086 BC patients’ data from the TCGA database were collected to verify the results from Kaplan–Meier plotter database.

### 2.4 cBioPortal analysis

Based on cBioPortal [23] (https://www.cbioportal.org) database, we chose the Invasive Breast Carcinoma database (METABRIC, Nature 2012, and Nat Commun 2016) to explore and construct the BC genome atlas of the SFXN family.

### 2.5 STRING analysis

STRING [24] (https://string-db.org) database was used to establish an SFXN family protein network to explore the potential function of the SFXN family.

### 2.6 DNA methylation analysis

MethSurv [25] (https://biit.cs.ut.ee/methsurv/) database is a web tool that can perform multivariable survival analysis using DNA methylation data. We used MethSurv database to explore the relationship between SFXN family methylation sites and prognostic value in BC patients.

### 2.7 TISIDB analysis

TISIDB [26] (http://cis.hku.hk/TISIDB/) database is a web portal for tumour and immune system interaction, which integrates multiple heterogeneous data types. Using TISIDB database, we analysed the relations between abundance of tumour-infiltrating lymphocytes and SFXN1 and SFXN2 expression. In addition, distributions of SFXN1 and SFXN2 expression across immune and molecular subtypes were also shown in our research.

### 2.8 ENCORI analysis

Using ENCORI [27] (http://starbase.sysu.edu.cn/panCancer.php) database, we predicted some miRNAs that regulated SFXN1 and SFXN2 expression in BC. Analysis parameters were medium stringency (≥3) and one cancer type.

### 2.9 Statistical analysis

Using Student’s *t*-tests, the statistical difference between the SFXN family mRNA expression level in BC and normal samples was revealed. Based on log-rank test and hazard ratio (HR), Kaplan–Meier plotter was used to draft survival curves that could reveal the relation between the expression of SFXN family and prognostic value of BC patients. *P* < 0.05 was considered to be statistically significant. Statistical analysis and visualization were done using R-project (version 3.6.3).

## 3 Results

### 3.1 Transcriptional levels of SFXN family in BC patients

**Figure 1:**
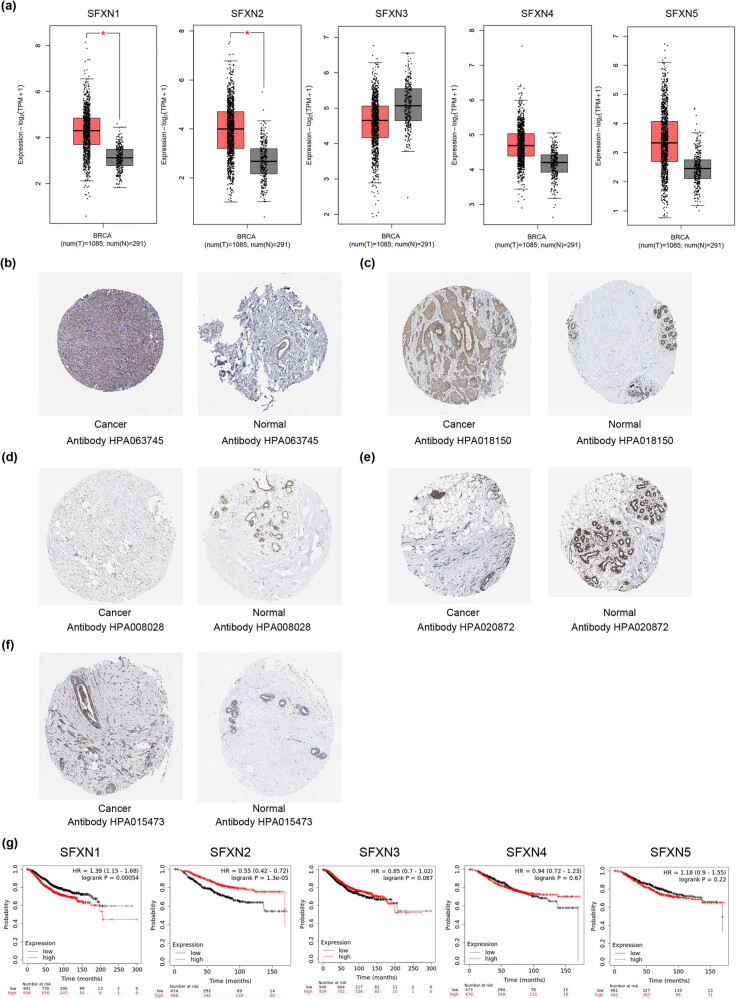
The transcriptional levels of SFXN1–SFXN5 in BC. (a) The transcriptional levels of SFXN1–SFXN5 were analysed based on GEPIA2 database. *P < 0.05. (b) SFXN1 (antibody: HPA063745) and (c) SFXN2 (antibody: HPA018150) were overexpressed in BC tissues versus normal breast tissues by immunohistochemistry based on HPA database, but not (d) SFXN3 (antibody: HPA008208), (e) SFXN4 (antibody: HPA020872), and (f) SFXN5 (antibody: HPA015473). (g) Prognostic value of the expression levels of SFXN1–SFXN5 in BC patients based on Kaplan–Meier plotter.

Using GEPIA2 database, the transcriptional levels of the SFXN family were analysed to reveal the statistical difference between the SFXN family mRNA expression level in BC and normal samples. We found that SFXN1 and SFXN2 mRNA transcriptional levels were significantly upregulated in BC samples compared with normal samples, but not SFXN3–SFXN5 (Figure 1a). Furthermore, based on the immunohistochemistry results from HPA database, SFXN1 (antibody: HPA063745) and SFXN2 (antibody: HPA018150) were overexpressed in BC tissues versus normal breast tissues (Figure 1b and c). The expression levels of SFXN3 (antibody: HPA008208), SFXN4 (antibody: HPA020872), and SFXN5 (antibody: HPA015473) in BC tissues were not significantly upregulated compared to normal breast tissues (Figure 1d–f).

### 3.2 Prognostic value of SFXN family in clinical patients with BC

Using Kaplan–Meier plotter, we explored the impact of the SFXN family expression on the overall survival (OS) rate of BC patients. As shown in Figure 1g, upregulated mRNA expression levels of SFXN1 were significantly related to poor prognosis in BC patients (HR = 1.39, *P* = 0.00054) but increased SFXN2 expression was significantly associated with good prognosis in patients with BC (HR = 0.55, *P* = 1.3 × 10^−5^). We did not find the relationships between SFXN3-5 expression and prognosis in BC patients. A TCGA cohort of 1086 BC patients also verified the above-mentioned results (Figure S1).

### 3.3 Alterations, correlations, and potential role analysis among SFXN family members

**Figure 2:**
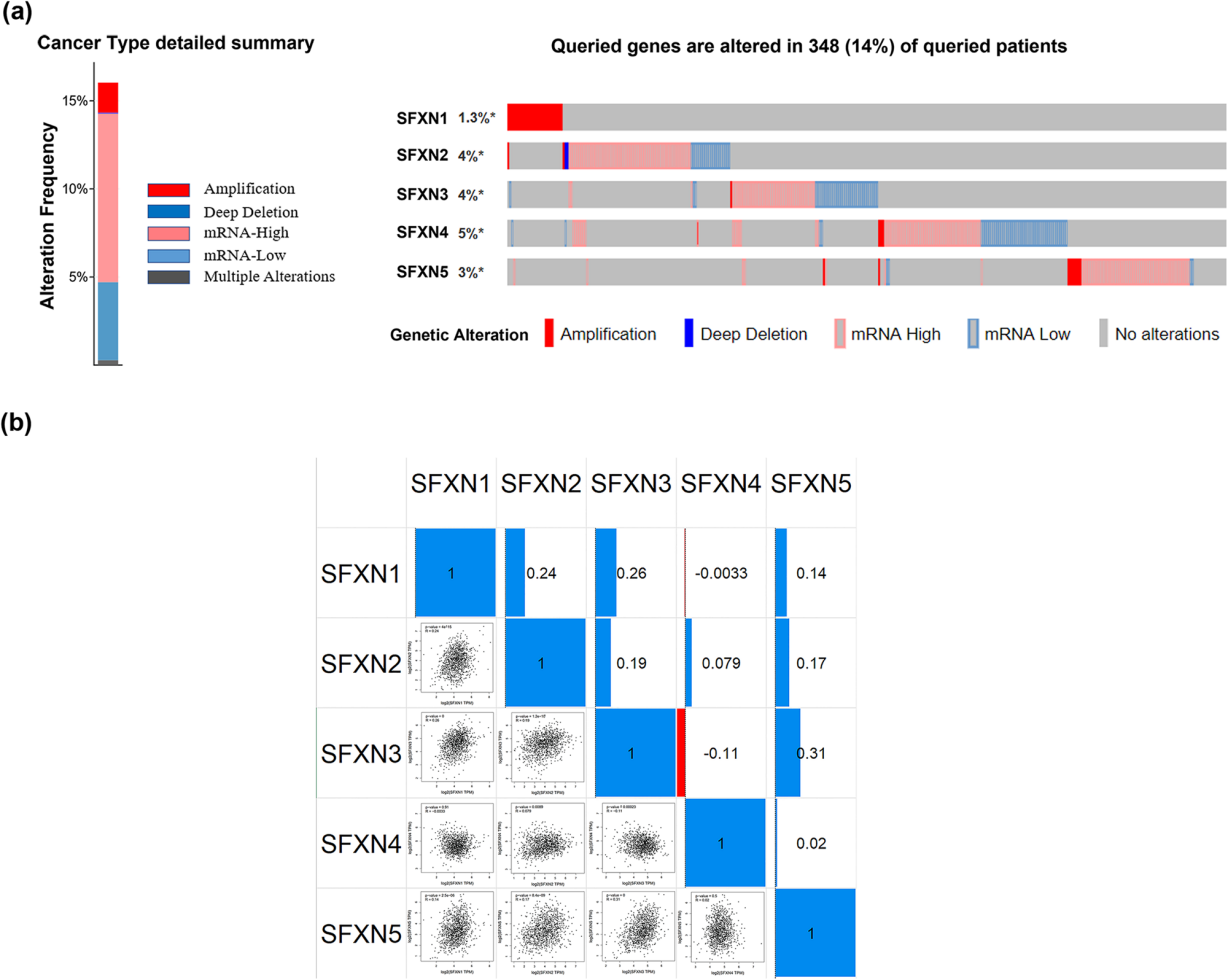
Alterations and correlation between members of SFXN family in BC. (a) SFXN family expression and alterations in BC based on cBioPortal. (b) Correlation analysis between SFXN family members in BC based on GEPIA.

We detected the alterations of SFXN1–SFXN5 in BC by the cBioPortal database. We found that SFXN1–SFXN5 were altered in 348 of the 2509 BC samples (14%), including amplification, deep deletion, high mRNA expression, and low mRNA expression (Figure 2a). Using the GEPIA2 database, the correlations of SFXN family members in BC were analysed. As shown in Figure 2b, the results were as follows: SFXN1–SFXN2 (*R* = 0.24), SFXN1–SFXN3 (*R* = 0.26), SFXN1–SFXN4 (*R* = −0.0033), SFXN1–SFXN5 (*R* = 0.14), SFXN2–SFXN3 (*R* = 0.19), SFXN2–SFXN4 (*R* = 0.079), SFXN2–SFXN5 (*R* = 0.17), SFXN3–SFXN4 (*R* = −0.11), SFXN3–SFXN5 (*R* = 0.31), and SFXN4–SFXN5 (*R* = 0.02).

Furthermore, in order to explore the potential function of the SFXN family, Gene Ontology (GO) analysis for the SFXN family was performed to predict the potential functions of the SFXN family based on STRING database, including biological processes (BPs), cellular components, and molecular functions. We found that GO:1990542 (mitochondrial transmembrane transport), GO:0006730 (one-carbon metabolic process), GO:0006865 (amino acid transport), GO:0022889 (serine transmembrane transporter activity), GO:0046943 (carboxylic acid transmembrane transporter activity), GO:0015075 (ion transmembrane transporter activity), and GO:0031305 (integral component of mitochondrial inner membrane) were significantly regulated by alterations in the SFXN family (Table 1). The important roles of the SFXN family in mitochondrial transmembrane transport and one-carbon metabolic process have been widely recognized.

**Table 1:** Functional annotation enrichment analysis of SFXN family performed by STRING database

	GO term	Count	False discovery rate (FDR)
	GO:1990542 (mitochondrial transmembrane transport)	5	4.05 × 10^−8^
BP	GO:0006730 (one-carbon metabolic process)	2	0.0288
	GO:0006865 (amino acid transport)	5	9.67 × 10^−8^
	GO:0022889 (serine transmembrane transporter activity)	3	5.60 × 10^−6^
MF	GO:0046943 (carboxylic acid transmembrane transporter activity)	4	3.29 × 10^−5^
	GO:0015075 (ion transmembrane transporter activity)	5	9.26 × 10^−5^
CC	GO:0031305 (integral component of mitochondrial inner membrane)	5	2.04 × 10^−10^

### 3.4 Relationship between SFXN family methylation sites and prognostic value in BC patients

**Figure 3:**
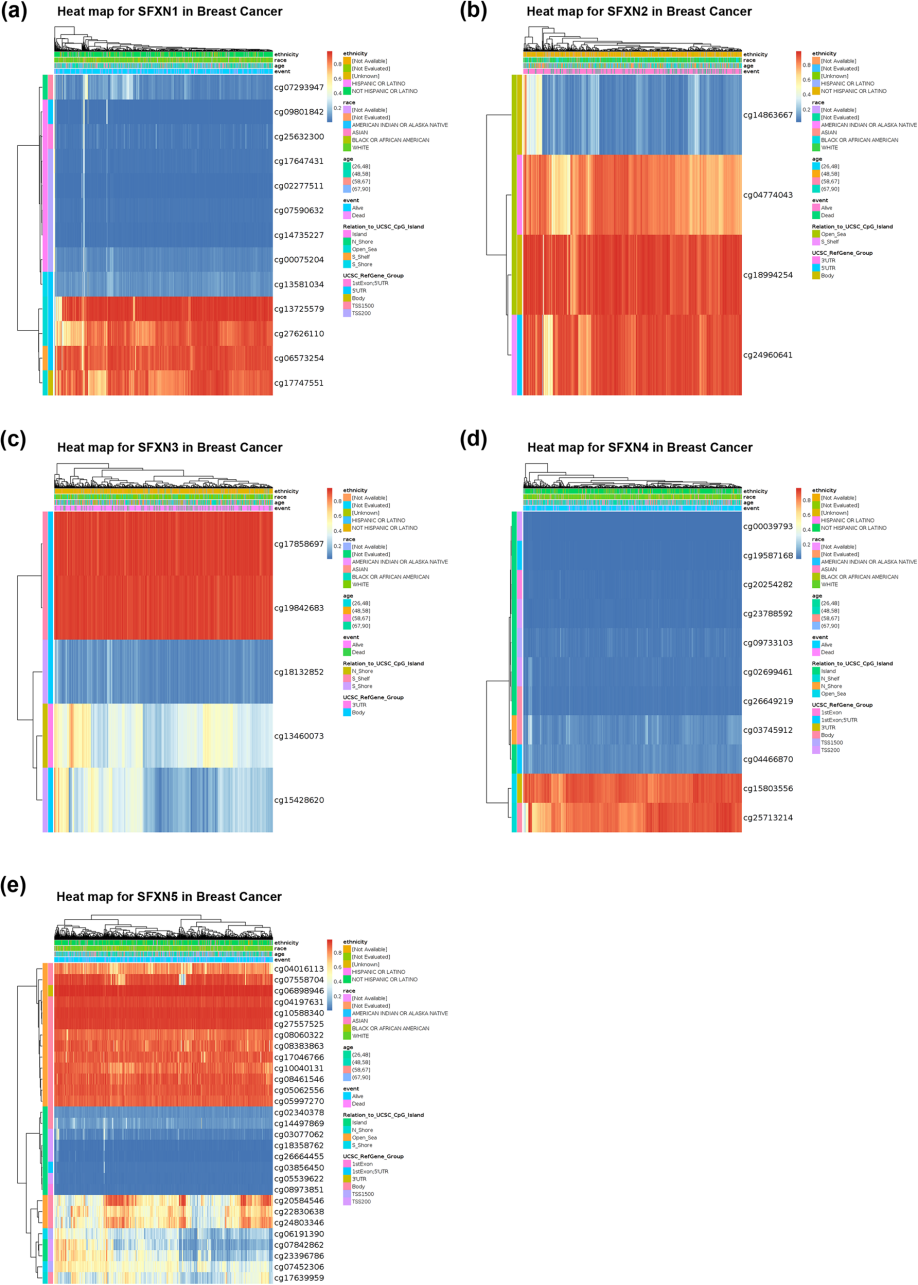
The relationship between SFXN1 and SFXN2 methylation sites and prognostic value in BC patients. (a) Heat map for SFXN1 in BC. (b) Heat map for SFXN2 in BC. (c) Heat map for SFXN3 in BC. (d) Heat map for SFXN4 in BC. (e) Heat map for SFXN5 in BC.

MethSurv database was used to perform multivariable survival analysis to reveal the relationship between SFXN1 and SFXN2 methylation sites and prognosis in BC patients. The methylation profiles of SFXN1 and SFXN2 were obtained from MethSurv database. We found that 13 SFXN1 CpG sites (Figure 3a), of which 2 CpG sites (5′-UTR-S_Shelf-cg06573254 and TSS200-Island-cg17647431) were significantly related to the prognosis of BC patients (Table 2). Four SFXN2 CpG sites (Figure 3b) were found, of which two CpG sites (3′-UTR-Open_Sea-cg04774043 and Body-Open_Sea-cg18994254) were significantly associated with the prognosis of BC patients (Table 3). Five SFXN3 CpG sites (Figure 3c) were found, of which one CpG site (Body-S_Shelf-cg17858697) was significantly related to the prognosis of BC patients (Table 4). Moreover, we found that 29 SFXN5 CpG sites (Figure 3e), of which 9 CpG sites (5′-UTR-S_Shelf-cg06573254 and TSS200-Island-cg17647431) were significantly associated with the prognosis of BC patients (Table 5). However, we did not find correlations between SFXN4 CpG sites and prognosis in BC patients (Figure 3d and Table 6).

**Table 2:** Effect of hypermethylation level of SFXN1 on prognosis in BC

CpG	HR	CI	*P* Value
TSS200-Island-cg00075204	0.804	(0.505–1.28)	0.357016246
TSS200-Island-cg02277511	1.218	(0.763–1.945)	0.408550216
**5**′**-UTR-S_Shelf-cg06573254**	**0.587**	**(0.359–0.958)**	**0.033011463**
TSS1500-N_Shore-cg07293947	0.758	(0.472–1.216)	0.249926515
TSS200-Island-cg07590632	0.8	(0.486–1.318)	0.381746405
5′-UTR-Island-cg09801842	1.176	(0.749–1.847)	0.481301929
5′-UTR-S_Shore-cg13581034	0.663	(0.437–1.006)	0.053534213
5′-UTR-Open_Sea-cg13725579	0.723	(0.491–1.066)	0.10171772
TSS200-Island-cg14735227	0.7	(0.436–1.123)	0.139380396
**TSS200-Island-cg17647431**	**0.477**	**(0.318–0.715)**	**0.000337326**
Body-Open_Sea-cg17747551	1.529	(0.945–2.683)	0.080503652
(1stExon;5′-UTR)-Island-cg25632300	0.701	(0.455–1.082)	0.108483031
5′-UTR-Open_Sea-cg27626110	1.157	(0.756–1.772)	0.501969845

Bold values are used for information with *P* < 0.05.

**Table 3:** Effect of hypermethylation level of SFXN2 on prognosis in BC

CpG	HR	CI	*P* Value
**3**′**-UTR-Open_Sea-cg04774043**	**0.597**	**(0.403–0.885)**	**0.010100815**
Body-Open_Sea-cg14863667	0.674	(0.446–1.021)	0.062516563
**Body-Open_Sea-cg18994254**	**0.495**	**(0.332–0.736)**	**0.000527837**
5′-UTR-S_Shelf-cg24960641	0.838	(0.53–1.324)	0.448366312

Bold values are used for information with *P* < 0.05.

**Table 4:** Effect of hypermethylation level of SFXN3 on prognosis in BC

CpG	HR	CI	*P* Value
3′-UTR-N_Shore-cg13460073	1.137	(0.773–1.674)	0.513879699
Body-S_Shore-cg15428620	0.664	(0.403–1.093)	0.107383131
**Body-S_Shelf-cg17858697**	**0.407**	**(0.227–0.73)**	**0.002523129**
Body-S_Shore-cg18132852	0.635	(0.385–1.046)	0.0745156
Body-S_Shelf-cg19842683	1.469	(0.817–2.64)	0.19858941

Bold values are used for information with *P* < 0.05.

**Table 5:** Effect of hypermethylation level of SFXN5 on prognosis in BC

CpG	HR	CI	*P* Value
Body-Island-cg02340378	0.763	(0.476–1.224)	0.261905244
TSS200-Island-cg03077062	1.106	(0.75–1.632)	0.610669504
**(1stExon;5**′**-UTR)-Island-cg03856450**	**0.63**	**(0.419–0.948)**	**0.026834993**
**Body-Open_Sea-cg04016113**	**0.385**	**(0.219–0.677)**	**0.000916438**
**Body-Open_Sea-cg04197631**	**0.638**	**(0.432–0.943)**	**0.024000916**
Body-Open_Sea-cg05062556	1.565	(0.916–2.673)	0.101212683
TSS200-Island-cg05539622	0.737	(0.477–1.139)	0.169875526
Body-Open_Sea-cg05997270	1.492	(0.874–2.548)	0.142590859
TSS1500-S_Shore-cg06191390	1.075	(0.667–1.731)	0.767367535
3′-UTR-Open_Sea-cg06898946	1.565	(0.917–2.669)	0.100221362
TSS1500-S_Shore-cg07452306	1.247	(0.811–1.917)	0.314599158
**Body-Open_Sea-cg07558704**	**0.549**	**(0.326–0.925)**	**0.024167969**
TSS200-Island-cg07842862	0.629	(0.373–1.059)	0.080831914
Body-Open_Sea-cg08060322	0.731	(0.443–1.203)	0.217589883
**Body-Open_Sea-cg08383863**	**0.497**	**(0.291–0.849)**	**0.010445105**
Body-Open_Sea-cg08461546	1.117	(0.757–1.646)	0.577394194
1stExon-Island-cg08973851	1.265	(0.806–1.987)	0.306727837
**Body-Open_Sea-cg10040131**	**1.85**	**(1.083–3.159)**	**0.02423507**
**Body-Open_Sea-cg10588340**	**0.566**	**(0.336–0.954)**	**0.032586494**
Body-Island-cg14497869	1.383	(0.908–2.108)	0.131331579
**Body-Open_Sea-cg17046766**	**1.604**	**(1.035–2.485)**	**0.034460369**
Body-N_Shore-cg17639959	0.78	(0.509–1.193)	0.251790437
TSS200-Island-cg18358762	0.772	(0.522–1.141)	0.19455617
Body-Open_Sea-cg20584546	1.199	(0.811–1.771)	0.362740545
**Body-Open_Sea-cg22830638**	**1.497**	**(1.006–2.228)**	**0.046907355**
TSS200-Island-cg23396786	0.853	(0.535–1.358)	0.502214225
Body-Open_Sea-cg24803346	1.328	(0.891–1.979)	0.163122628
TSS200-Island-cg26664455	0.877	(0.594–1.293)	0.506775452
Body-Open_Sea-cg27557525	1.347	(0.887–2.046)	0.161840548

Bold values are used for information with *P* < 0.05.

**Table 6:** Effect of hypermethylation level of SFXN4 on prognosis in BC

CpG	HR	CI	*P* Value
TSS200-Island-cg00039793	1.537	(0.961–2.459)	0.072648992
TSS200-Island-cg02699461	0.595	(0.349–1.015)	0.056815652
Body-N_Shore-cg03745912	1.301	(0.826–2.05)	0.256731252
(1stExon;5′-UTR)-Island-cg04466870	0.85	(0.571–1.267)	0.424668793
TSS1500-Island-cg09733103	0.669	(0.423–1.057)	0.084919658
3′-UTR-Open_Sea-cg15803556	0.765	(0.518–1.13)	0.17824569
(1stExon;5′-UTR)-Island-cg19587168	1.124	(0.762–1.657)	0.556229052
1stExon-Island-cg20254282	1.259	(0.793–1.998)	0.329115199
TSS200-Island-cg23788592	0.879	(0.556–1.388)	0.579432626
Body-N_Shelf-cg25713214	0.692	(0.428–1.118)	0.132244808
Body-Island-cg26649219	1.106	(0.751–1.628)	0.611426216

### 3.5 SFXN1 and SFXN2 expression levels are associated with immune and molecular subtypes and immune cell infiltration in BC

Next, we explored the roles of the SFXN1 and SFXN2 expression on immune and molecular subtypes in BC based on TISIDB database. The immune subtypes were classified into six types, namely, C1 (wound healing), C2 (IFN-gamma dominant), C3 (inflammatory), C4 (lymphocyte depleted), C5 (immunologically quiet), and C6 (TGF-b dominant). Our results showed that the SFXN1 and SFXN2 expression levels were significantly related to immune subtypes in BC (Figure S2a and b). Moreover, we also found that the SFXN1 and SFXN2 expression levels were significantly associated with molecular subtypes in BC (Figure S2c and d).

In addition, the relationships between abundance of tumour-infiltrating lymphocytes and SFXN1 and SFXN2 expression levels are given in Tables 7 and 8. We found that SFXN1 expression was significantly related to activated B cell (*R* = −0.323, *P* = 2.2 × 10^−16^), and SFXN2 expression was significantly associated with effector memory CD8T cell (*R* = 0.328, *P* = 2.2 × 10^−16^), T follicular helper cell (*R* = −0.393, *P* = 2.2 × 10^−16^), gamma delta T cell (*R* = −0.335, *P* = 2.2 × 10^−16^), type 1 T helper cell (*R* = −0.378, *P* = 2.2 × 10^−16^), regulatory T cell (*R* = −0.31, *P* = 2.2 × 10^−16^), myeloid-derived suppressor cell (*R* = −0.307, *P* = 2.2 × 10^−16^), natural killer T cell (*R* = −0.354, *P* = 2.2 × 10^−16^), and activated dendritic cell (*R* = −0.325, *P* = 2.2 × 10^−16^). These results indicated that SFXN1 and SFXN2 expression levels played essential roles in the immune cell infiltration in BC.

**Table 7:** Relations between abundance of tumour-infiltrating lymphocytes and SFXN1 expression in breast cancer

Description	Correlation	*P* value
Activated CD8T cell	−0.149	7.08 × 10^−7^
Central memory CD8T cell	0.065	0.0308
Effector memory CD8T cell	−0.256	8.11 × 10^−18^
Activated CD4T cell	0.057	0.0579
Central memory CD8T cell	−0.244	2.46 × 10^−16^
Effector memory CD8T cell	0.022	0.459
T follicular helper cell	−0.223	8.33 × 10^−14^
Gamma delta T cell	−0.196	5.45 × 10^−11^
Type 1 T helper cell	−0.285	5.63 × 10^−22^
Type 17 T helper cell	−0.235	3.93 × 10^−15^
Type 2 T helper cell	−0.02	0.498
Regulatory T cell	−0.113	0.000183
**Activated B cell**	**−0.323**	**2.2** × 10^−**16**^
Immature B cell	−0.149	7.67 × 10^−7^
Memory B cell	−0.051	0.0923
Natural killer cell	−0.171	1.18 × 10^−8^
CD56natural killer cell	−0.269	1.5 × 10^−19^
CD56dim killer cell	−0.198	4.08 × 10^−11^
Myeloid-derived suppressor cell	−0.192	1.48 × 10^−10^
Natural killer T cell	−0.282	1.75 × 10^−21^
Activated dendritic cell	−0.06	0.0466
Plasmacytoid dendritic cell	−0.212	1.38 × 10^−12^
Immature dendritic cell	−0.011	0.717
Macrophage	−0.208	3.93 × 10^−12^
Eosinophil	−0.173	8.95 × 10^−9^
Mast cell	−0.292	4.31 × 10^−31^
Monocyte	−0.167	2.52 × 10^−8^
Neutrophil	−0.11	0.000249

Bold values are used for information with *P* < 0.05.

**Table 8:** Relations between abundance of tumour-infiltrating lymphocytes and SFXN2 expression in breast cancer

Description	Correlation	*P* value
Activated CD8T cell	−0.212	1.42 × 10^−12^
Central memory CD8T cell	−0.018	0.541
**Effector memory CD8T cell**	**0.328**	**2.2** × 10^−**16**^
Activated CD4T cell	−0.254	1.48 × 10^−17^
Central memory CD4T cell	−0.263	9.54 × 10^−19^
Effector memory CD4T cell	−0.142	2.31 × 10^−6^
**T follicular helper cell**	**−0.393**	**2.2** × 10^−**16**^
**Gamma delta T cell**	**−0.335**	**2.2** × 10^−**16**^
**Type 1 T helper cell**	**−0.378**	**2.2** × 10^−**16**^
Type 17 T helper cell	−0.234	5.18 × 10^−15^
Type 2 T helper cell	−0.246	1.49 × 10^−16^
**Regulatory T cell**	**−0.31**	**2.2** × 10^−**16**^
Activated B cell	−0.252	2.61 × 10^−17^
Immature B cell	−0.189	3.06 × 10^−10^
Memory B cell	−0.177	3.67 × 10^−9^
Natural killer cell	−0.133	1 × 10^−5^
CD56 natural killer cell	−0.219	2.63 × 10^−13^
CD56dim killer cell	−0.264	6.29 × 10^−19^
**Myeloid-derived suppressor cell**	**−0.307**	**2.2** × 10^−**16**^
**Natural killer T cell**	**−0.354**	**2.2** × 10^−**16**^
**Activated dendritic cell**	**−0.325**	**2.2** × 10^−**16**^
Plasmacytoid dendritic cell	−0.153	3.73 × 10^−7^
Immature dendritic cell	0.023	0.439
Macrophage	−0.218	2.82 × 10^−13^
Eosinophil	−0.075	0.0123
Mast cell	−0.204	1.02 × 10^−11^
Monocyte	−0.219	2.13 × 10^−13^
Neutrophil	−0.045	0.135

Bold values are used for information with *P* < 0.05.

### 3.6 Immune cell-type expression and expression cluster analysis for SFXN1 and SFXN2

**Figure 4:**
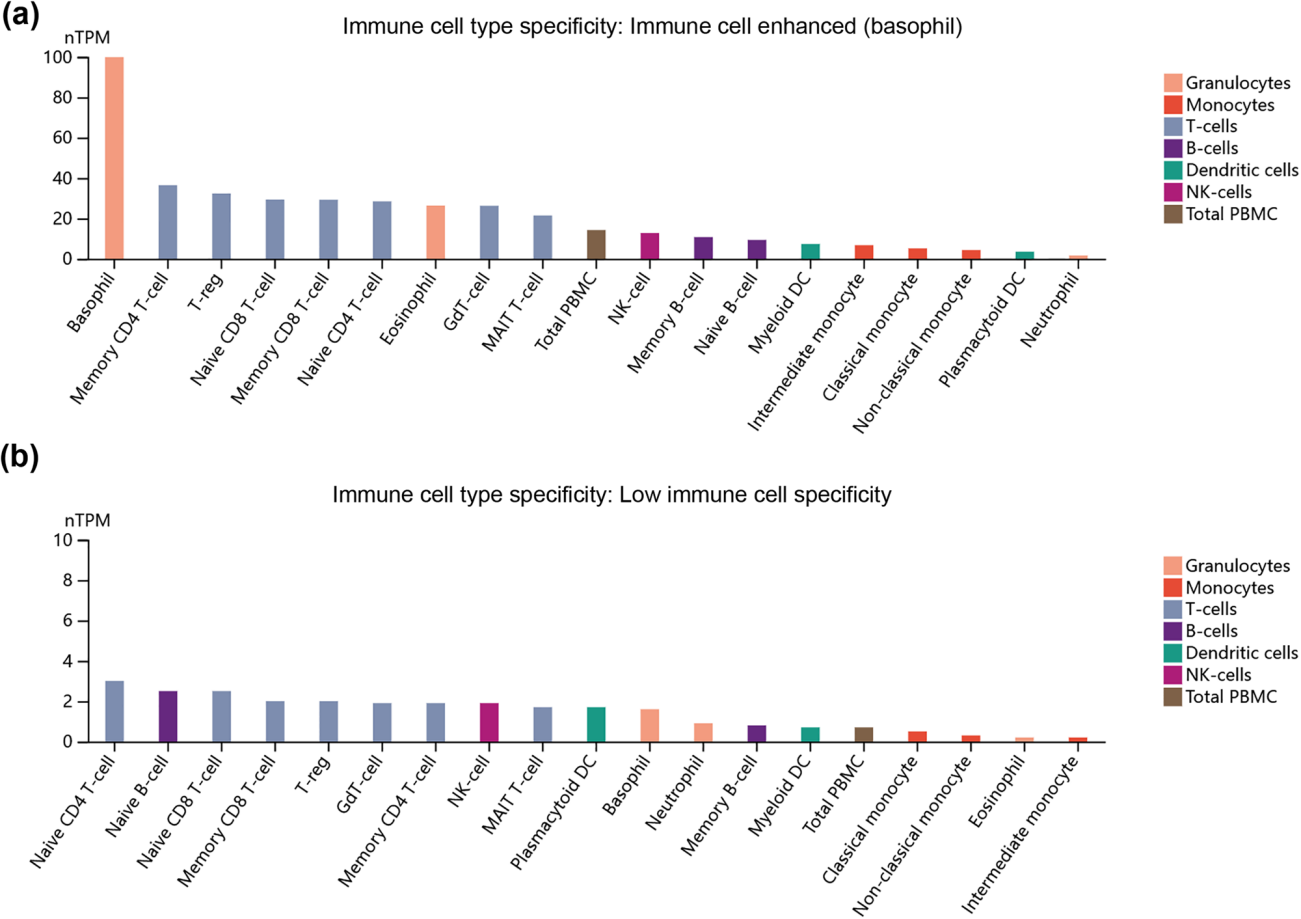
Expression of immune cell types and expression cluster analysis for SFXN1 and SFXN2. (a) SFXN1 was predominantly expressed in basophils. (b) SFXN2 was low immune cell specificity.

Furthermore, we explored the expression of immune cell types and expression clusters for SFXN1 and SFXN2. We found that SFXN1 was predominantly expressed in basophils (Figure 4a) and was a part of cluster basophil–DNA binding. Ten nearest neighbour genes significantly related to SFXN1 in basophil–DNA binding clusters were identified based on immune cell RNA expression, namely, FBLN5, KLHL35, ENPP2, RAB3GAP2, CCDC126, HRH4, HPGD, CD244, C10orf82, and CACNA1D (Table 9). Moreover, we also found that SFXN2 was low immune cell specificity (Figure 4b) and was a part of the T cell–unknown function cluster. Ten nearest neighbour genes significantly associated with SFXN2 in T cell–unknown function cluster were identified based on immune cell RNA expression, namely, ZNF563, SLC9A3, CFAP70, C14orf132, METAP1D, FAM153A, TRABD2A, ST3GAL3, REV1, and WNK3 (Table 10). As shown in Figure S3a, FBLN5, KLHL35, ENPP2, and HPGD were significantly overexpressed in BC compared with normal samples. However, we did not find any gene significantly associated with SFXN2 in the T cell–unknown function cluster, which was significantly upregulated in BC versus normal samples (Figure S3b). Furthermore, our results indicated that high expression of ENPP2, FBLN5, HPGD, and KLHL35 was significantly related to good prognosis for BC patients (Figure S4).

**Table 9:** Ten nearest neighbour genes significantly related to SFXN1 in Basophil–DNA binding cluster based on immune cell RNA expression

Neighbour	Description	Correlation
FBLN5	Fibulin 5	0.7909
KLHL35	Kelch-like family member 35	0.7869
ENPP2	Ectonucleotide pyrophosphatase/phosphodiesterase 2	0.7815
RAB3GAP2	RAB3 GTPase-activating noncatalytic protein subunit 2	0.7780
CCDC126	Coiled-coil domain containing 126	0.7691
HRH4	Histamine receptor H4	0.7664
HPGD	15-hydroxyprostaglandin dehydrogenase	0.7628
CD244	CD244 molecule	0.7562
C10orf82	Chromosome 10 open reading frame 82	0.7544
CACNA1D	Calcium voltage-gated channel subunit alpha1 D	0.7504

**Table 10:** Ten nearest neighbour genes significantly related to SFXN2 in T cells–unknown function cluster based on immune cell RNA expression

Neighbour	Description	Correlation
ZNF563	Zinc finger protein 563	0.7651
SLC9A3	Solute carrier family 9 member A3	0.7642
CFAP70	Cilia and flagella-associated protein 70	0.7482
C14orf132	Chromosome 14 open reading frame 132	0.7433
METAP1D	Methionyl aminopeptidase type 1D, mitochondrial	0.7143
FAM153A	Family with sequence similarity 153 member A	0.7077
TRABD2A	TraB domain containing 2A	0.7010
ST3GAL3	ST3 beta-galactoside alpha-2,3-sialyltransferase 3	0.6877
REV1	REV1 DNA-directed polymerase	0.6872
WNK3	WNK lysine-deficient protein kinase 3	0.6694

### 3.7 Regulatory miRNA analysis for SFXN1 and SFXN2 in BC

**Figure 5:**
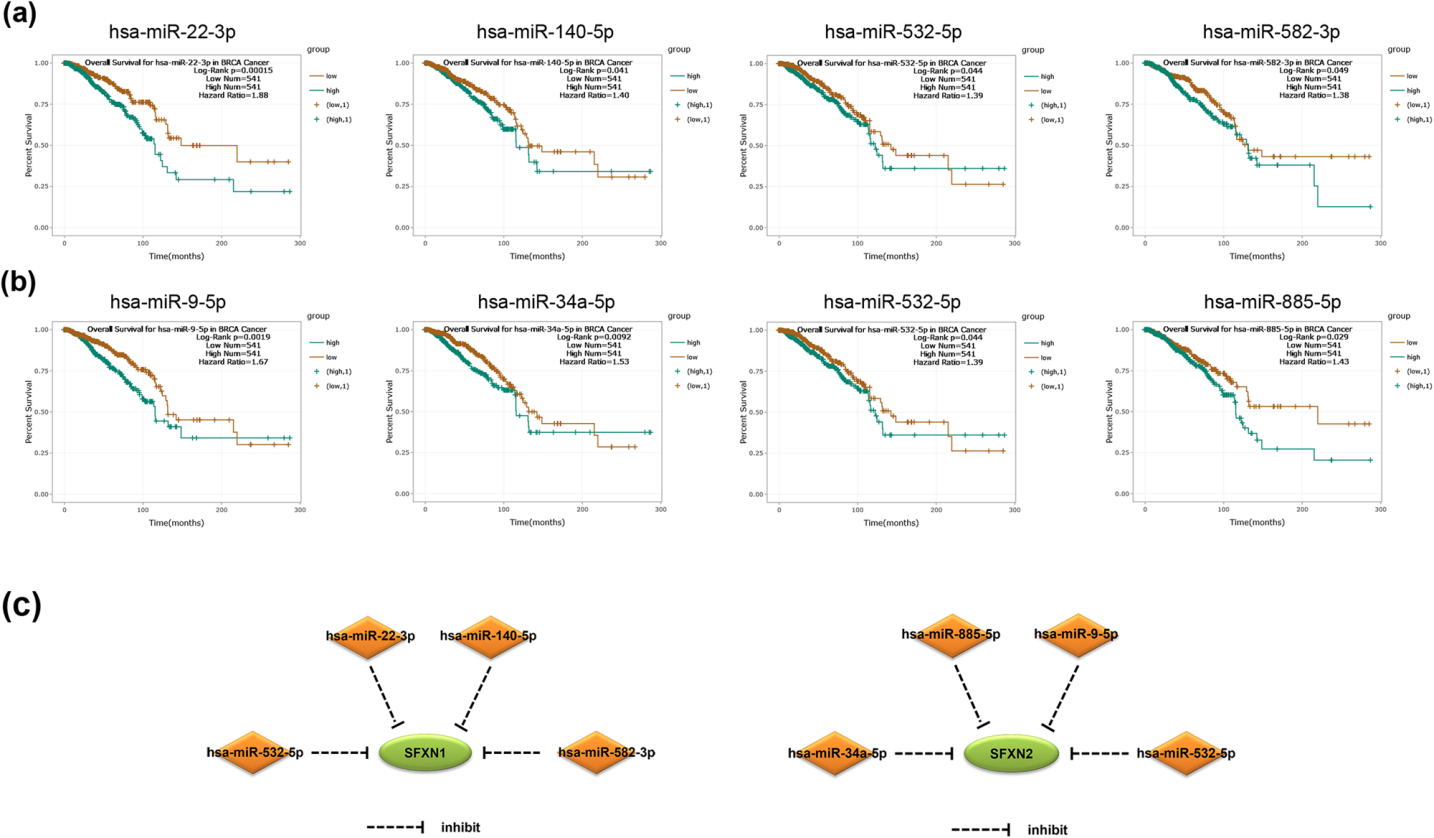
The miRNA expression in BC predicted to regulate SFXN1 and SFXN2 and related to the prognosis of BC patients. (a) has-miR-22-3p, has-miR-140-5p, has-miR-532-5p, and has-miR-582-3p were related to poor OS in BC patients. (b) has-miR-9-5p, has-miR-34a-5p, has-miR-532-5p, and has-miR-885-5p were associated with poor OS in BC patients. (c) miRNA–SFXN1 and miRNA–SFXN2 regulation networks.

As shown in Tables S1 and S2, 173 miRNAs regulating SFXN1 and 97 miRNAs regulating SFXN2 were predicted based on ENCORI database. Among them, 132 miRNA–SFXN1 pairs and 79 miRNA–SFXN2 pairs were negatively correlated. As shown in Figure 5a, four negative regulatory miRNA–SFXN1 pairs were significantly related to poor prognosis for BC patients, namely, has-miR-22-3p (HR = 1.88, *P* = 0.00015) [28], has-miR-140-5p (HR = 1.40, *P* = 0.041), has-miR-532-5p (HR = 1.39, *P* = 0.044), and has-miR-582-3p (HR = 1.38, *P* = 0.049). Moreover, as shown in Figure 5b, four negative regulatory miRNA–SFXN2 pairs were significantly associated with poor prognosis for BC patients and miRNAs negatively regulated SFXN2, namely, has-miR-9-5p (HR = 1.67, *P* = 0.0019), has-miR-34a-5p (HR = 1.53, *P* = 0.0092), has-miR-532-5p (HR = 1.39, *P* = 0.044), and has-miR-885-5p (HR = 1.48, *P* = 0.029). The above-mentioned results suggest that the established miRNA–SFXN1 and miRNA–SFXN2 regulatory networks may be valuable prognostic markers and therapeutic targets for BC (Figure 5c).

## 4 Discussion

At present, the role of the SFXN family in BC has not been clearly reported. Using bioinformatics tools, this study systematically explored different aspects of the SFXN family in BC, including expression, prognosis, methylation, immune infiltration, and regulatory miRNA analysis. We found that SFXN1 and SFXN2 mRNA transcriptional levels were significantly upregulated in BC, but the same was not true of SFXN3–SFXN5. High SFXN1 expression was significantly related to poor prognosis in BC patients, but increased SFXN2 expression was significantly associated with good prognosis in patients with BC. Moreover, some CpG sites of the SFXN family were associated with the prognosis of BC patients. It has been reported that SFXN1 is overexpressed in head and neck squamous cell carcinoma and that high SFXN1 expression in this context is significantly associated with poor prognosis [29]. Jiang et al. reported that upregulated SFXN1 expression is significantly related to poor prognosis in lung adenocarcinoma patients [30]. In addition, it has been reported that SFXN2 is present in four different cancer types, namely, kidney cancer, urothelial cancer, cervical cancer, and liver cancer; only for kidney cancer is SFXN2 favourable [31].

Next, we performed GO analysis of the SFXN family to predict its potential functions. We found that the GO terms such as mitochondrial transmembrane transport, one-carbon metabolic process, amino acid transport, serine transmembrane transporter activity, carboxylic acid transmembrane transporter activity, ion transmembrane transporter activity, and integral component of mitochondrial inner membrane were significantly regulated by alterations in the SFXN family. The roles of SFXN1 and SFXN2 in mitochondria have been widely recognized. SFXN1 plays an important role in mitochondrial single-carbon metabolism, and SFXN1-mediated serine transport from the cytoplasm to mitochondria can be promoted by SERAC1 [10,32]. Moreover, SFXN1 deficiency causes damage to the mitochondrial respiratory chain, compromising CIII biosynthesis [11]. SFXN2 is a mitochondrial outer membrane protein that plays a role in mitochondrial iron homeostasis by regulating haem biosynthesis [12]. In addition, Pott et al. reported that SFXN2 expression was able to affect plaque burden in carotid arteries [33].

Furthermore, we explored the expression in immune cell types and expression clusters for SFXN1 and SFXN2. We found that SFXN1 was predominantly expressed in basophils and was a part of the basophil–DNA binding cluster. Four nearest neighbor genes significantly associated with SFXN1 in the basophil–DNA binding cluster were identified, namely, FBLN5, KLHL35, ENPP2, and HPGD, which were significantly overexpressed in BC compared with normal samples and related to good prognosis in BC patients. It has been reported that FBLN5 inhibits the proliferation and invasion of BC cells by downregulating Ki-67 [34]. Mao et al. reported that hsa-miR-370-3p promoted BC progression by inhibiting FBLN5 expression and activating the NF-κB signalling pathway [35]. KLHL35 is highly expressed in colon cancer tissues compared with normal samples and is associated with poor prognosis in colon cancer patients [36]. ENPP2 autocrine motility factor expression levels can be promoted by integrin alpha6beta4 in BC cells [37]. Lehtinen et al. reported that HPGD was highly expressed in metastatic and invasive BCs [38]. However, we did not find any gene significantly associated with SFXN2 in the T cell–unknown function cluster, which was significantly upregulated in BC versus normal samples.

MicroRNA expression is related to tumour occurrence, progression, and therapy, indicating that microRNAs may be useful as prognostic and predictive markers [39]. We identified eight negatively correlated miRNA–SFXN1 and miRNA–SFXN2 pairs, namely hsa-miR-22-3p–SFXN1, hsa-miR-140-5p–SFXN1, hsa-miR-582-3p–SFXN1, hsa-miR-532-5p–SFXN1, hsa-miR-9-5p–SFXN2, hsa-miR-34a-5p–SFXN2, hsa-miR-532-5p–SFXN2, and hsa-miR-885-5p–SFXN2, which might regulate SFXN1 and SFXN2 expression in BC and were used to explore the prognosis of BC patients. Functionally, decreased expression of hsa-miR-22-3p was related to good prognosis for BC patients [28]. It has been reported that hsa-miR-140-5p inhibits the proliferation of BC stem cells by targeting Wnt1 [40]. Huang et al. reported that hsa-miR-532-5p promoted BC proliferation and migration by targeting ras-related and oestrogen-regulated growth inhibitor [41]. The expression level of hsa-miR-9-5p is significantly increased in the serum of BC patients versus healthy controls and predicts poor prognosis for BC patients [42]. Maroni et al. reported that hsa-miR-34a-5p was upregulated in nonmetastatic breast carcinoma [43]. It has been reported that hsa-miR-885-5p can be a potential predictor of anthracycline-induced troponin elevation in BC patients [44]. However, the role of hsa-miR-582-3p in BC has not been reported. Our research could enrich the prognostic value of miRNAs regulating SFXN1 and SFXN2 in BC, which would be helpful for the discovery of BC markers and precise treatment targets.

## 5 Conclusion

In this study, we systematically analysed the expression levels, functions, and prognostic values of SFXN family in BC. Our results suggest that elevated SFXN1 and SFXN2 play essential roles in BC. High SFXN1 expression was significantly related to poor prognosis in BC patients, and high SFXN2 expression was significantly associated with good prognosis in patients with BC. Moreover, we found that two SFXN1 CpG sites (5′-UTR-S_Shelf-cg06573254 and TSS200-Island-cg17647431), two SFXN2 CpG sites (3′-UTR-Open_Sea-cg04774043 and Body-Open_Sea-cg18994254), one SFXN3 CpG site (Body-S_Shelf-cg17858697), nine SFXN5 CpG sites (1stExon; 5′-UTR-Island-cg03856450, Body-Open_Sea-cg04016113, Body-Open_Sea-cg04197631, Body-Open_Sea-cg07558704, Body-Open_Sea-cg08383863, Body-Open_Sea-cg10040131, Body-Open_Sea-cg10588340, Body-Open_Sea-cg17046766, and Body-Open_Sea-cg22830638) were significantly related to the prognosis of BC patients. The miRNAs regulating SFXN1 (has-miR-22-3p, has-miR-140-5p, has-miR-532-5p, and has-miR-582-3p regulating SFXN1) and SFXN2 (has-miR-9-5p, has-miR-34a-5p, has-miR-532-5p, and has-miR-885-5p regulating SFXN2) could be involved in carcinogenesis and influencing the prognosis of BC patients. The detailed studies on mechanisms of SFXN family related to the occurrence, progression, and treatment for BC are required to explore new therapeutic strategies for patients with BC.

## Supplementary Material

Supplementary material
