# Supplementary material for: Comprehensive analysis of the role of SFXN family in breast cancer
Source: Open Med (Wars). 2023 Apr 1;18(1):20230685. doi: 10.1515/med-2023-0685 (PMC10068752; doi:10.1515/med-2023-0685)
Supplement: Supplementary material [file med-2023-0685-sm.pdf]

# Supplementary material

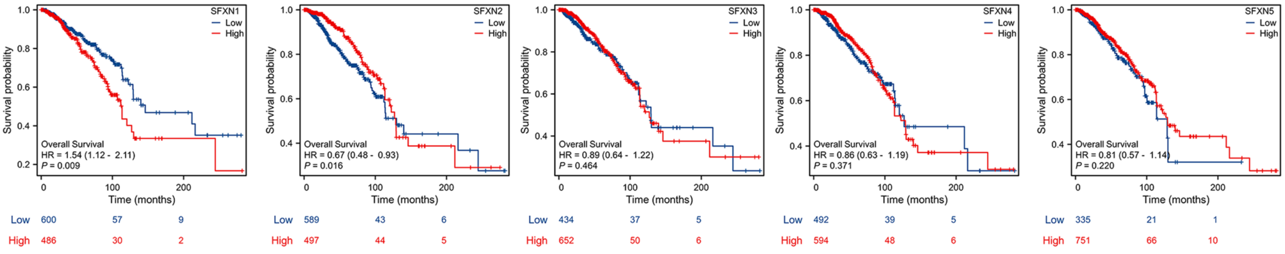


**Figure S1:** The survival analysis results from the Kaplan-Meier plotter were validated through a TCGA cohort of 1086 breast cancer patients. (a) High SFXN1 expression was significantly related to poor prognosis in BC patients. (b) High SFXN2 expression was significantly associated with good prognosis in BC patients. We did not find the relationships between the expression of (c) SFXN3, (d) SFXN4, and (e) SFXN5 and prognosis in BC patients.

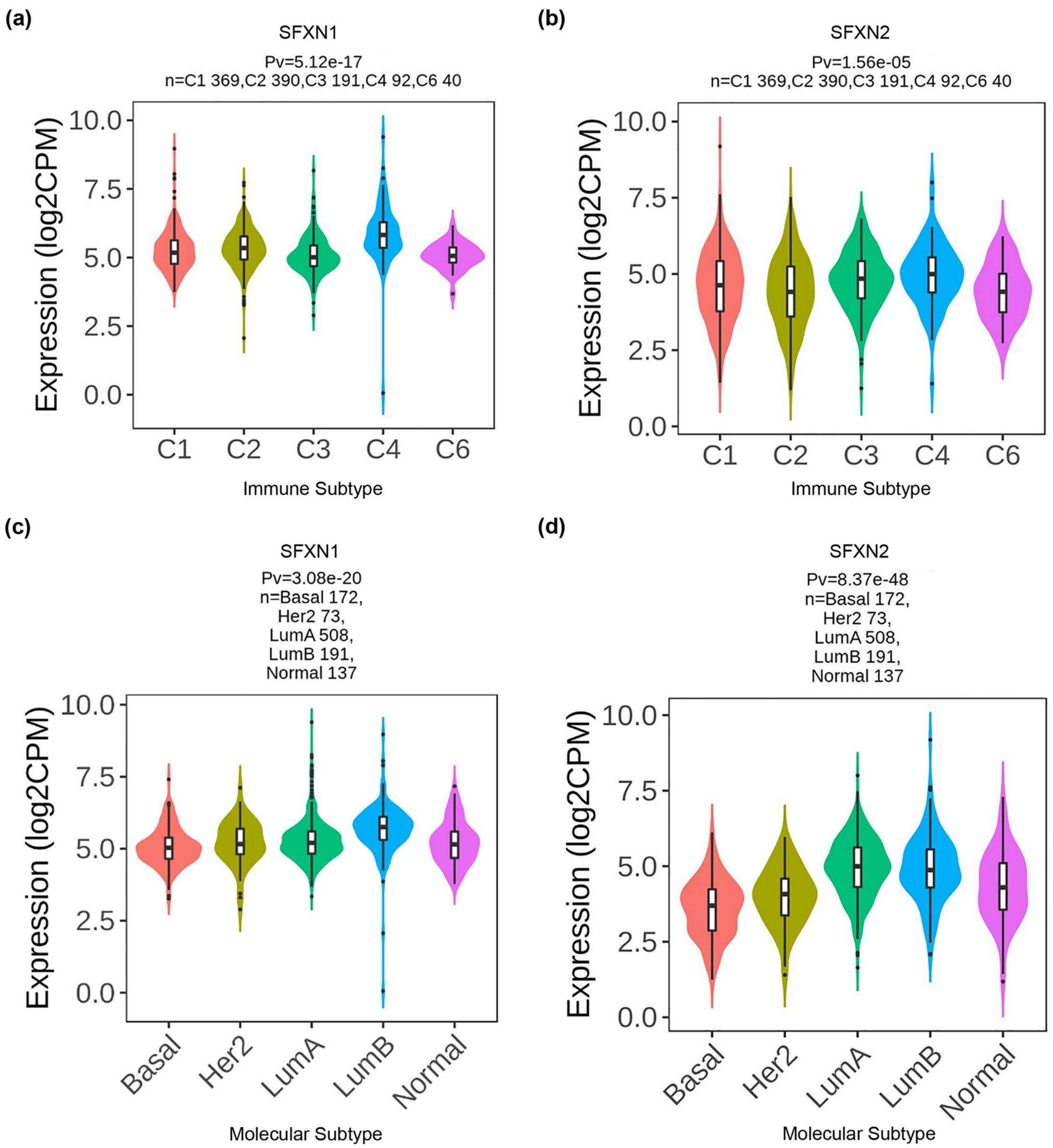


**Figure S2:** The SFXN1 and SFXN2 expression are related to immune and molecular subtypes in BC. Distribution of (a) SFXN1 and (b) SFXN2 expression across immune subtype. Distribution of (c) SFXN1 and (d) SFXN2 expression across molecular subtype.

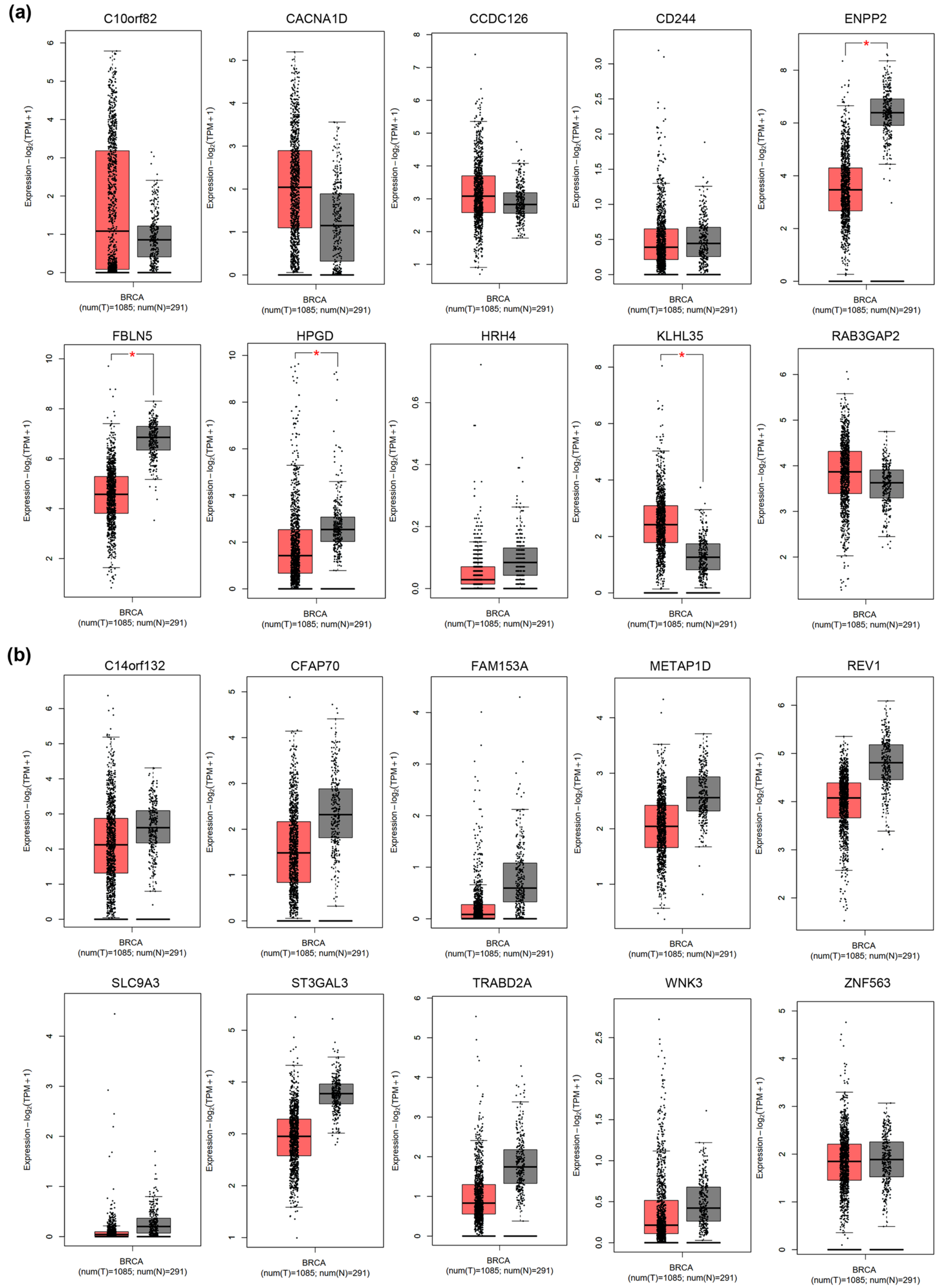


**Figure S3:** The transcriptional levels analysis of neighbor genes significantly associated with SFXN1 and SFXN2 in BC based on immune cell RNA expression. (a) The transcriptional levels of ten nearest neighbor genes significantly related to SFXN1 in basophils - DNA binding cluster. (b) The transcriptional levels of ten nearest neighbor genes significantly associated with SFXN2 in T-cells - Unknown function cluster.

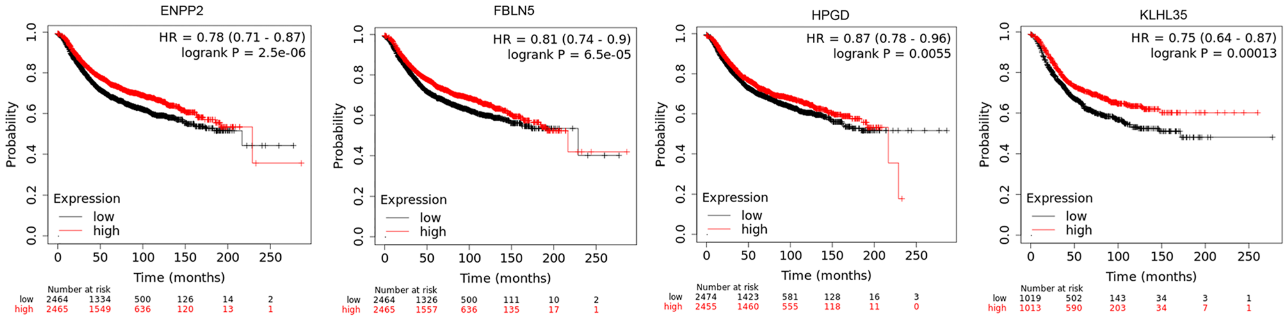


**Figure S4:** Prognostic value of the expression levels of ENPP2, FBLN5, HPGD and KLHL35 in BC patients based on Kaplan-Meier Plotter.

**Table S1:** Correlation of miRNA-SFXN1 pairs identified by ENCORI database

| No. | miRNA | Coefficient-R | *p*-Value |
|---|---|---|---|
| 1 | hsa-miR-16-5p | 0.020 | $5.13 \times 10^{-1}$ |
| 2 | hsa-miR-92a-3p | 0.021 | $4.94 \times 10^{-1}$ |
| 3 | hsa-miR-29b-3p | 0.124 | $4.02 \times 10^{-5}$ |
| 4 | hsa-miR-139-5p | 0.005 | $8.80 \times 10^{-1}$ |
| 5 | hsa-miR-212-3p | 0.031 | $3.15 \times 10^{-1}$ |
| 6 | hsa-miR-15b-5p | 0.041 | $1.77 \times 10^{-1}$ |
| 7 | hsa-miR-124-3p | 0.060 | $4.66 \times 10^{-2}$ |
| 8 | hsa-miR-132-3p | 0.041 | $1.82 \times 10^{-1}$ |
| 9 | hsa-miR-141-3p | 0.045 | $1.43 \times 10^{-1}$ |
| 10 | hsa-miR-200c-3p | 0.052 | $8.86 \times 10^{-2}$ |
| 11 | hsa-miR-29c-3p | 0.139 | $4.40 \times 10^{-6}$ |
| 12 | hsa-miR-375 | 0.087 | $4.10 \times 10^{-3}$ |
| 13 | hsa-miR-328-3p | 0.13 | $1.72 \times 10^{-5}$ |
| 14 | hsa-miR-342-3p | 0.159 | $1.31 \times 10^{-7}$ |
| 15 | hsa-miR-148b-3p | 0.106 | $4.81 \times 10^{-4}$ |
| 16 | hsa-miR-324-3p | 0.08 | $8.45 \times 10^{-3}$ |
| 17 | hsa-miR-338-3p | 0.239 | $1.40 \times 10^{-15}$ |
| 18 | hsa-miR-486-5p | 0.145 | $1.59 \times 10^{-6}$ |
| 19 | hsa-miR-490-3p | 0.019 | $5.21 \times 10^{-1}$ |
| 20 | hsa-miR-512-3p | 0.019 | $5.23 \times 10^{-1}$ |
| 21 | hsa-miR-498 | 0.011 | $7.14 \times 10^{-1}$ |
| 22 | hsa-miR-513a-5p | 0.020 | $5.16 \times 10^{-1}$ |
| 23 | hsa-miR-506-3p | 0.099 | $1.04 \times 10^{-3}$ |
| 24 | hsa-miR-92b-3p | 0.088 | $3.58 \times 10^{-3}$ |
| 25 | hsa-miR-425-5p | 0.046 | $1.31 \times 10^{-1}$ |
| 26 | hsa-miR-371a-5p | 0.020 | $5.16 \times 10^{-1}$ |
| 27 | hsa-miR-589-5p | 0.031 | $3.04 \times 10^{-1}$ |
| 28 | hsa-miR-628-5p | 0.032 | $2.91 \times 10^{-1}$ |
| 29 | hsa-miR-874-3p | 0.173 | $9.63 \times 10^{-9}$ |
| 30 | hsa-miR-1224-5p | 0.190 | $2.53 \times 10^{-10}$ |
| 31 | hsa-miR-513b-5p | 0.056 | $6.43 \times 10^{-2}$ |
| 32 | hsa-miR-513c-5p | 0.073 | $1.66 \times 10^{-2}$ |
| 33 | hsa-miR-1179 | 0.008 | $7.89 \times 10^{-1}$ |
| 34 | hsa-miR-1249-3p | 0.046 | $1.30 \times 10^{-1}$ |
| 35 | hsa-miR-1251-5p | 0.095 | $1.65 \times 10^{-3}$ |
| 36 | hsa-miR-1913 | 0.031 | $3.14 \times 10^{-1}$ |
| 37 | hsa-miR-514b-5p | 0.025 | $4.19 \times 10^{-1}$ |
| 38 | hsa-miR-3619-5p | 0.050 | $9.81 \times 10^{-2}$ |
| 39 | hsa-miR-3194-3p | 0.062 | $4.14 \times 10^{-2}$ |
| 40 | hsa-miR-506-5p | 0.003 | $9.32 \times 10^{-1}$ |

**Table S1:** *continued*

| No. | miRNA | Coefficient-R | *p*-Value |
|---|---|---|---|
| 41 | hsa-miR-374c-3p | 0.047 | $1.22 \times 10^{-1}$ |
| 42 | hsa-miR-15a-5p | −0.064 | $3.60 \times 10^{-2}$ |
| 43 | hsa-miR-22-3p | −0.074 | $1.49 \times 10^{-2}$ |
| 44 | hsa-miR-23a-3p | −0.05 | $9.83 \times 10^{-2}$ |
| 45 | hsa-miR-25-3p | −0.039 | $2.01 \times 10^{-1}$ |
| 46 | hsa-miR-26a-5p | −0.088 | $3.73 \times 10^{-3}$ |
| 47 | hsa-miR-26b-5p | −0.037 | $2.29 \times 10^{-1}$ |
| 48 | hsa-miR-27a-3p | −0.086 | $4.41 \times 10^{-3}$ |
| 49 | hsa-miR-28-5p | −0.187 | $5.03 \times 10^{-10}$ |
| 50 | hsa-miR-29a-3p | −0.052 | $8.94 \times 10^{-2}$ |
| 51 | hsa-miR-30a-5p | −0.042 | $1.72 \times 10^{-1}$ |
| 52 | hsa-miR-31-5p | −0.109 | $3.21 \times 10^{-4}$ |
| 53 | hsa-miR-32-5p | −0.013 | $6.65 \times 10^{-1}$ |
| 54 | hsa-miR-105-5p | −0.022 | $4.68 \times 10^{-1}$ |
| 55 | hsa-miR-196a-5p | −0.053 | $8.15 \times 10^{-2}$ |
| 56 | hsa-miR-197-3p | −0.049 | $1.08 \times 10^{-1}$ |
| 57 | hsa-miR-199a-5p | −0.128 | $2.26 \times 10^{-5}$ |
| 58 | hsa-miR-148a-3p | −0.153 | $3.77 \times 10^{-7}$ |
| 59 | hsa-miR-30c-5p | −0.077 | $1.08 \times 10^{-2}$ |
| 60 | hsa-miR-30d-5p | −0.042 | $1.63 \times 10^{-1}$ |
| 61 | hsa-miR-199b-5p | −0.157 | $1.92 \times 10^{-7}$ |
| 62 | hsa-miR-205-5p | −0.139 | $4.10 \times 10^{-6}$ |
| 63 | hsa-miR-214-3p | −0.112 | $2.22 \times 10^{-4}$ |
| 64 | hsa-miR-216a-5p | −0.072 | $1.85 \times 10^{-2}$ |
| 65 | hsa-miR-217 | −0.116 | $1.37 \times 10^{-4}$ |
| 66 | hsa-miR-221-3p | −0.06 | $4.67 \times 10^{-2}$ |
| 67 | hsa-miR-222-3p | −0.002 | $9.49 \times 10^{-1}$ |
| 68 | hsa-miR-224-5p | −0.153 | $4.03 \times 10^{-7}$ |
| 69 | hsa-miR-200b-3p | −0.014 | $6.41 \times 10^{-1}$ |
| 70 | hsa-miR-1-3p | −0.017 | $5.78 \times 10^{-1}$ |
| 71 | hsa-miR-23b-3p | −0.060 | $4.87 \times 10^{-2}$ |
| 72 | hsa-miR-27b-3p | −0.125 | $3.75 \times 10^{-5}$ |
| 73 | hsa-miR-30b-5p | −0.053 | $8.05 \times 10^{-2}$ |
| 74 | hsa-miR-122-5p | −0.018 | $5.58 \times 10^{-1}$ |
| 75 | hsa-miR-128-3p | −0.027 | $3.83 \times 10^{-1}$ |
| 76 | hsa-miR-135a-5p | −0.003 | $9.09 \times 10^{-1}$ |
| 77 | hsa-miR-137 | −0.072 | $1.83 \times 10^{-2}$ |
| 78 | hsa-miR-138-5p | −0.119 | $8.63 \times 10^{-5}$ |
| 79 | hsa-miR-140-5p | −0.089 | $3.19 \times 10^{-3}$ |
| 80 | hsa-miR-142-5p | −0.038 | $2.15 \times 10^{-1}$ |
| 81 | hsa-miR-143-3p | −0.057 | $6.05 \times 10^{-2}$ |

(*Continued*)

**Table S1:** *continued*

| No. | miRNA | Coefficient-R | *p*-Value |
|---|---|---|---|
| 82 | hsa-miR-145-5p | −0.096 | $1.61 \times 10^{-3}$ |
| 83 | hsa-miR-152-3p | −0.022 | $4.67 \times 10^{-1}$ |
| 84 | hsa-miR-134-5p | −0.070 | $2.14 \times 10^{-2}$ |
| 85 | hsa-miR-146a-5p | −0.110 | $2.89 \times 10^{-4}$ |
| 86 | hsa-miR-188-5p | −0.068 | $2.48 \times 10^{-2}$ |
| 87 | hsa-miR-195-5p | −0.125 | $3.60 \times 10^{-5}$ |
| 88 | hsa-miR-206 | −0.083 | $6.39 \times 10^{-3}$ |
| 89 | hsa-miR-320a | −0.048 | $1.17 \times 10^{-1}$ |
| 90 | hsa-miR-200a-3p | −0.001 | $9.67 \times 10^{-1}$ |
| 91 | hsa-miR-299-3p | −0.055 | $6.97 \times 10^{-2}$ |
| 92 | hsa-miR-30e-5p | −0.1 | $1.02 \times 10^{-3}$ |
| 93 | hsa-miR-361-5p | −0.116 | $1.22 \times 10^{-4}$ |
| 94 | hsa-miR-363-3p | −0.044 | $1.44 \times 10^{-1}$ |
| 95 | hsa-miR-369-3p | −0.083 | $6.47 \times 10^{-3}$ |
| 96 | hsa-miR-374a-5p | −0.148 | $1.06 \times 10^{-6}$ |
| 97 | hsa-miR-380-3p | −0.02 | $5.09 \times 10^{-1}$ |
| 98 | hsa-miR-381-3p | −0.101 | $9.07 \times 10^{-4}$ |
| 99 | hsa-miR-323a-3p | −0.077 | $1.07 \times 10^{-2}$ |
| 100 | hsa-miR-135b-5p | −0.141 | $3.16 \times 10^{-6}$ |
| 101 | hsa-miR-345-5p | −0.025 | $4.05 \times 10^{-1}$ |
| 102 | hsa-miR-196b-5p | −0.046 | $1.30 \times 10^{-1}$ |
| 103 | hsa-miR-424-5p | −0.052 | $8.66 \times 10^{-2}$ |
| 104 | hsa-miR-429 | −0.014 | $6.56 \times 10^{-1}$ |
| 105 | hsa-miR-433-3p | −0.044 | $1.52 \times 10^{-1}$ |
| 106 | hsa-miR-329-3p | −0.043 | $1.55 \times 10^{-1}$ |
| 107 | hsa-miR-410-3p | −0.070 | $2.18 \times 10^{-2}$ |
| 108 | hsa-miR-483-3p | −0.015 | $6.22 \times 10^{-1}$ |
| 109 | hsa-miR-485-3p | −0.075 | $1.30 \times 10^{-2}$ |
| 110 | hsa-miR-487a-3p | −0.053 | $7.93 \times 10^{-2}$ |
| 111 | hsa-miR-489-3p | −0.061 | $4.31 \times 10^{-2}$ |
| 112 | hsa-miR-146b-5p | −0.127 | $2.70 \times 10^{-5}$ |
| 113 | hsa-miR-493-5p | −0.065 | $3.27 \times 10^{-2}$ |
| 114 | hsa-miR-494-3p | −0.097 | $1.39 \times 10^{-3}$ |
| 115 | hsa-miR-495-3p | −0.119 | $8.56 \times 10^{-5}$ |
| 116 | hsa-miR-496 | −0.057 | $6.23 \times 10^{-2}$ |
| 117 | hsa-miR-497-5p | −0.085 | $4.90 \times 10^{-3}$ |
| 118 | hsa-miR-515-5p | −0.018 | $5.49 \times 10^{-1}$ |
| 119 | hsa-miR-519e-5p | −0.003 | $9.28 \times 10^{-1}$ |
| 120 | hsa-miR-520f-3p | −0.002 | $9.51 \times 10^{-1}$ |
| 121 | hsa-miR-503-5p | −0.024 | $4.31 \times 10^{-1}$ |

**Table S1:** *continued*

| No. | miRNA | Coefficient-R | *p*-Value |
|---|---|---|---|
| 122 | hsa-miR-505-3p | −0.113 | $1.82 \times 10^{-4}$ |
| 123 | hsa-miR-514a-3p | −0.009 | $7.65 \times 10^{-1}$ |
| 124 | hsa-miR-532-5p | −0.139 | $4.00 \times 10^{-6}$ |
| 125 | hsa-miR-556-5p | −0.055 | $6.86 \times 10^{-2}$ |
| 126 | hsa-miR-576-5p | −0.038 | $2.13 \times 10^{-1}$ |
| 127 | hsa-miR-579-3p | −0.052 | $8.99 \times 10^{-2}$ |
| 128 | hsa-miR-590-5p | −0.073 | $1.68 \times 10^{-2}$ |
| 129 | hsa-miR-641 | −0.019 | $5.29 \times 10^{-1}$ |
| 130 | hsa-miR-655-3p | −0.080 | $8.27 \times 10^{-3}$ |
| 131 | hsa-miR-656-3p | −0.058 | $5.62 \times 10^{-2}$ |
| 132 | hsa-miR-542-3p | −0.113 | $1.82 \times 10^{-4}$ |
| 133 | hsa-miR-758-3p | −0.089 | $3.38 \times 10^{-3}$ |
| 134 | hsa-miR-28-3p | −0.206 | $7.26 \times 10^{-12}$ |
| 135 | hsa-miR-362-3p | −0.135 | $8.52 \times 10^{-6}$ |
| 136 | hsa-miR-379-3p | −0.065 | $3.36 \times 10^{-2}$ |
| 137 | hsa-miR-340-5p | −0.070 | $2.09 \times 10^{-2}$ |
| 138 | hsa-miR-488-3p | −0.064 | $3.58 \times 10^{-2}$ |
| 139 | hsa-miR-545-5p | −0.022 | $4.64 \times 10^{-1}$ |
| 140 | hsa-miR-582-3p | −0.108 | $3.76 \times 10^{-4}$ |
| 141 | hsa-miR-411-3p | −0.078 | $1.05 \times 10^{-2}$ |
| 142 | hsa-miR-450b-5p | −0.07 | $2.09 \times 10^{-2}$ |
| 143 | hsa-miR-889-3p | −0.142 | $2.53 \times 10^{-6}$ |
| 144 | hsa-miR-876-5p | −0.002 | $9.40 \times 10^{-1}$ |
| 145 | hsa-miR-708-5p | −0.094 | $1.97 \times 10^{-3}$ |
| 146 | hsa-miR-665 | −0.059 | $5.10 \times 10^{-2}$ |
| 147 | hsa-miR-873-5p | −0.022 | $4.63 \times 10^{-1}$ |
| 148 | hsa-miR-543 | −0.069 | $2.39 \times 10^{-2}$ |
| 149 | hsa-miR-374b-5p | −0.11 | $2.67 \times 10^{-4}$ |
| 150 | hsa-miR-216b-5p | −0.016 | $5.94 \times 10^{-1}$ |
| 151 | hsa-miR-320b | −0.076 | $1.24 \times 10^{-2}$ |
| 152 | hsa-miR-320c | −0.085 | $4.87 \times 10^{-3}$ |
| 153 | hsa-miR-1323 | −0.023 | $4.48 \times 10^{-1}$ |
| 154 | hsa-miR-1298-5p | −0.016 | $5.97 \times 10^{-1}$ |
| 155 | hsa-miR-1287-5p | −0.017 | $5.81 \times 10^{-1}$ |
| 156 | hsa-miR-1294 | −0.018 | $5.51 \times 10^{-1}$ |
| 157 | hsa-miR-548o-3p | −0.025 | $4.06 \times 10^{-1}$ |
| 158 | hsa-miR-320d | −0.06 | $4.81 \times 10^{-2}$ |
| 159 | hsa-miR-3139 | −0.024 | $4.30 \times 10^{-1}$ |
| 160 | hsa-miR-3140-3p | −0.055 | $7.22 \times 10^{-2}$ |
| 161 | hsa-miR-3163 | −0.037 | $2.18 \times 10^{-1}$ |

(*Continued*)

**Table S1:** *continued*

| No. | miRNA | Coefficient-R | *p*-Value |
|---|---|---|---|
| 162 | hsa-miR-3171 | −0.028 | $3.58 \times 10^{-1}$ |
| 163 | hsa-miR-3622a-5p | −0.051 | $9.63 \times 10^{-2}$ |
| 164 | hsa-miR-3622b-5p | −0.006 | $8.37 \times 10^{-1}$ |
| 165 | hsa-miR-374c-5p | −0.020 | $5.08 \times 10^{-1}$ |
| 166 | hsa-miR-4429 | −0.025 | $4.04 \times 10^{-1}$ |
| 167 | hsa-miR-4524a-5p | −0.036 | $2.40 \times 10^{-1}$ |
| 168 | hsa-miR-4677-3p | −0.064 | $3.37 \times 10^{-2}$ |
| 169 | hsa-miR-4770 | −0.022 | $4.68 \times 10^{-1}$ |
| 170 | hsa-miR-5579-3p | −0.081 | $7.90 \times 10^{-3}$ |
| 171 | hsa-miR-664b-3p | −0.006 | $8.34 \times 10^{-1}$ |
| 172 | hsa-miR-5586-5p | −0.024 | $4.39 \times 10^{-1}$ |
| 173 | hsa-miR-892c-5p | −0.047 | $1.22 \times 10^{-1}$ |

**Table S2:** Correlation of miRNA-SFXN2 pairs identified by ENCORI database

| No. | miRNA | Coefficient-R | *p*-Value |
|---|---|---|---|
| 1 | hsa-let-7b-5p | 0.145 | $1.63 \times 10^{-6}$ |
| 2 | hsa-miR-103a-3p | 0.102 | $7.34 \times 10^{-4}$ |
| 3 | hsa-miR-204-5p | 0.03 | $3.25 \times 10^{-1}$ |
| 4 | hsa-miR-195-5p | 0.064 | $3.50 \times 10^{-2}$ |
| 5 | hsa-miR-34c-5p | 0.124 | $3.94 \times 10^{-5}$ |
| 6 | hsa-miR-365a-3p | 0.024 | $4.31 \times 10^{-1}$ |
| 7 | hsa-miR-342-3p | 0.313 | $4.38 \times 10^{-26}$ |
| 8 | hsa-miR-20b-5p | 0.021 | $4.93 \times 10^{-1}$ |
| 9 | hsa-miR-449a | 0.241 | $9.50 \times 10^{-16}$ |
| 10 | hsa-miR-483-3p | 0.028 | $3.64 \times 10^{-1}$ |
| 11 | hsa-miR-491-5p | 0.062 | $3.97 \times 10^{-2}$ |
| 12 | hsa-miR-497-5p | 0.02 | $5.10 \times 10^{-1}$ |
| 13 | hsa-miR-449b-5p | 0.186 | $6.48 \times 10^{-10}$ |
| 14 | hsa-miR-760 | 0.09 | $2.90 \times 10^{-3}$ |
| 15 | hsa-miR-449c-5p | 0.187 | $4.93 \times 10^{-10}$ |
| 16 | hsa-miR-3612 | 0.005 | $8.73 \times 10^{-1}$ |
| 17 | hsa-miR-4524a-5p | 0.012 | $6.81 \times 10^{-1}$ |
| 18 | hsa-miR-4784 | 0.094 | $1.91 \times 10^{-3}$ |
| 19 | hsa-let-7c-5p | −0.135 | $8.24 \times 10^{-6}$ |
| 20 | hsa-let-7d-5p | −0.193 | $1.31 \times 10^{-10}$ |
| 21 | hsa-let-7e-5p | −0.02 | $5.03 \times 10^{-1}$ |
| 22 | hsa-let-7f-5p | −0.027 | $3.81 \times 10^{-1}$ |
| 23 | hsa-miR-15a-5p | −0.045 | $1.43 \times 10^{-1}$ |
| 24 | hsa-miR-16-5p | −0.05 | $1.01 \times 10^{-1}$ |
| 25 | hsa-miR-17-5p | −0.233 | $6.95 \times 10^{-15}$ |
| 26 | hsa-miR-20a-5p | −0.198 | $4.27 \times 10^{-11}$ |
| 27 | hsa-miR-24-3p | −0.375 | $1.23 \times 10^{-37}$ |
| 28 | hsa-miR-27a-3p | −0.328 | $1.24 \times 10^{-28}$ |
| 29 | hsa-miR-28-5p | −0.212 | $1.87 \times 10^{-12}$ |
| 30 | hsa-miR-33a-5p | −0.185 | $8.11 \times 10^{-10}$ |
| 31 | hsa-miR-93-5p | −0.097 | $1.45 \times 10^{-3}$ |
| 32 | hsa-miR-98-5p | −0.14 | $3.45 \times 10^{-6}$ |
| 33 | hsa-miR-106a-5p | −0.068 | $2.42 \times 10^{-2}$ |
| 34 | hsa-miR-107 | −0.021 | $4.91 \times 10^{-1}$ |
| 35 | hsa-miR-34a-5p | −0.159 | $1.32 \times 10^{-7}$ |
| 36 | hsa-miR-211-5p | −0.07 | $2.19 \times 10^{-2}$ |
| 37 | hsa-miR-212-3p | −0.193 | $1.34 \times 10^{-10}$ |
| 38 | hsa-miR-218-5p | −0.102 | $7.50 \times 10^{-4}$ |
| 39 | hsa-let-7g-5p | −0.12 | $7.57 \times 10^{-5}$ |
| 40 | hsa-let-7i-5p | −0.309 | $2.07 \times 10^{-25}$ |

**Table S2:** *continued*

| No. | miRNA | Coefficient-R | *p*-Value |
|---|---|---|---|
| 41 | hsa-miR-15b-5p | −0.104 | $6.16 \times 10^{-4}$ |
| 42 | hsa-miR-27b-3p | −0.2 | $2.74 \times 10^{-11}$ |
| 43 | hsa-miR-128-3p | −0.127 | $2.91 \times 10^{-5}$ |
| 44 | hsa-miR-132-3p | −0.165 | $5.00 \times 10^{-8}$ |
| 45 | hsa-miR-138-5p | −0.217 | $5.38 \times 10^{-13}$ |
| 46 | hsa-miR-9-5p | −0.379 | $2.50 \times 10^{-38}$ |
| 47 | hsa-miR-146a-5p | −0.229 | $2.04 \times 10^{-14}$ |
| 48 | hsa-miR-150-5p | −0.088 | $3.68 \times 10^{-3}$ |
| 49 | hsa-miR-154-5p | −0.096 | $1.52 \times 10^{-3}$ |
| 50 | hsa-miR-186-5p | −0.196 | $7.32 \times 10^{-11}$ |
| 51 | hsa-miR-155-5p | −0.245 | $2.38 \times 10^{-16}$ |
| 52 | hsa-miR-106b-5p | −0.158 | $1.73 \times 10^{-7}$ |
| 53 | hsa-miR-299-3p | −0.056 | $6.51 \times 10^{-2}$ |
| 54 | hsa-miR-380-3p | −0.065 | $3.35 \times 10^{-2}$ |
| 55 | hsa-miR-151a-3p | −0.104 | $6.01 \times 10^{-4}$ |
| 56 | hsa-miR-424-5p | −0.172 | $1.27 \times 10^{-8}$ |
| 57 | hsa-miR-329-3p | −0.083 | $6.19 \times 10^{-3}$ |
| 58 | hsa-miR-485-3p | −0.141 | $3.13 \times 10^{-6}$ |
| 59 | hsa-miR-146b-5p | −0.275 | $2.90 \times 10^{-20}$ |
| 60 | hsa-miR-494-3p | −0.052 | $8.90 \times 10^{-2}$ |
| 61 | hsa-miR-495-3p | −0.139 | $4.31 \times 10^{-6}$ |
| 62 | hsa-miR-498 | −0.051 | $9.24 \times 10^{-2}$ |
| 63 | hsa-miR-520a-5p | −0.053 | $7.91 \times 10^{-2}$ |
| 64 | hsa-miR-525-5p | −0.05 | $9.80 \times 10^{-2}$ |
| 65 | hsa-miR-519d-3p | −0.033 | $2.76 \times 10^{-1}$ |
| 66 | hsa-miR-503-5p | −0.113 | $2.01 \times 10^{-4}$ |
| 67 | hsa-miR-513a-5p | −0.016 | $6.06 \times 10^{-1}$ |
| 68 | hsa-miR-532-5p | −0.246 | $2.21 \times 10^{-16}$ |
| 69 | hsa-miR-545-3p | −0.089 | $3.27 \times 10^{-3}$ |
| 70 | hsa-miR-579-3p | −0.083 | $6.23 \times 10^{-3}$ |
| 71 | hsa-miR-580-3p | −0.105 | $5.33 \times 10^{-4}$ |
| 72 | hsa-miR-33b-5p | −0.029 | $3.36 \times 10^{-1}$ |
| 73 | hsa-miR-411-5p | −0.075 | $1.32 \times 10^{-2}$ |
| 74 | hsa-miR-660-5p | −0.193 | $1.55 \times 10^{-10}$ |
| 75 | hsa-miR-421 | −0.169 | $2.04 \times 10^{-8}$ |
| 76 | hsa-miR-361-3p | −0.114 | $1.65 \times 10^{-4}$ |
| 77 | hsa-miR-362-3p | −0.133 | $1.04 \times 10^{-5}$ |
| 78 | hsa-miR-532-3p | −0.246 | $1.77 \times 10^{-16}$ |
| 79 | hsa-miR-455-3p | −0.214 | $9.35 \times 10^{-13}$ |
| 80 | hsa-miR-589-5p | −0.088 | $3.81 \times 10^{-3}$ |

(*Continued*)

**Table S2:** *continued*

| No. | miRNA | Coefficient-R | *p*-Value |
|---|---|---|---|
| 81 | hsa-miR-708-5p | −0.19 | $2.54 \times 10^{-10}$ |
| 82 | hsa-miR-885-5p | −0.162 | $8.74 \times 10^{-8}$ |
| 83 | hsa-miR-665 | −0.081 | $7.67 \times 10^{-3}$ |
| 84 | hsa-miR-873-5p | −0.147 | $1.19 \times 10^{-6}$ |
| 85 | hsa-miR-944 | −0.017 | $5.81 \times 10^{-1}$ |
| 86 | hsa-miR-1224-5p | −0.043 | $1.54 \times 10^{-1}$ |
| 87 | hsa-miR-1296-5p | −0.06 | $4.79 \times 10^{-2}$ |
| 88 | hsa-miR-1323 | −0.009 | $7.69 \times 10^{-1}$ |
| 89 | hsa-miR-548o-3p | −0.095 | $1.68 \times 10^{-3}$ |
| 90 | hsa-miR-1269a | −0.126 | $2.95 \times 10^{-5}$ |
| 91 | hsa-miR-3139 | −0.007 | $8.08 \times 10^{-1}$ |
| 92 | hsa-miR-3681-3p | −0.06 | $4.70 \times 10^{-2}$ |
| 93 | hsa-miR-3150b-3p | −0.103 | $6.47 \times 10^{-4}$ |
| 94 | hsa-miR-4731-5p | −0.016 | $5.95 \times 10^{-1}$ |
| 95 | hsa-miR-4739 | −0.027 | $3.72 \times 10^{-1}$ |
| 96 | hsa-miR-4761-5p | −0.053 | $8.37 \times 10^{-2}$ |
| 97 | hsa-miR-766-5p | −0.02 | $5.17 \times 10^{-1}$ |
